# Cytoplasmic dynein1 intermediate-chain2 regulates cellular trafficking and physiopathological development in *Magnaporthe oryzae*

**DOI:** 10.1016/j.isci.2023.106050

**Published:** 2023-02-10

**Authors:** Lily Lin, Ibrahim Tijjani, Hengyuan Guo, Qiuli An, Jiaying Cao, Xiaomin Chen, Wende Liu, Zonghua Wang, Justice Norvienyeku

**Affiliations:** 1 Ministerial and Provincial Joint Innovation Centre for Safety Production of Cross-Strait Crops, Fujian Agriculture and Forestry University, Fuzhou 350002, China; 2 Key Laboratory of Green Prevention and Control of Tropical Plant Diseases and Pests, Ministry of Education, College of Plant Protection, Hainan University, Haikou, China; 3 State Key Laboratory for Biology of Plant Diseases and Insect Pests, Institute of Plant Protection, Chinese Academy of Agricultural Sciences, Beijing 100193, China; 4 Hainan Yazhou Bay Seed Laboratory, Sanya Nanfan Research Institute of Hainan University, Sanya, China; 5 Institute of Oceanography, Minjiang University, Fuzhou 350108, China

**Keywords:** Molecular microbiology, Cell biology, Molecular plant pathology

## Abstract

The cytoplasmic dynein 1, a minus end-directed motor protein, is an essential microtubule-based molecular motor that mediates the movement of molecules to intracellular destinations in eukaryotes. However, the role of dynein in the pathogenesis of *Magnaporthe oryzae* is unknown. Here, we identified cytoplasmic dynein 1 intermediate-chain 2 genes in *M. oryzae* and functionally characterized it using genetic manipulations, and biochemical approaches. We observed that targeted the deletion of *MoDYNC1I2* caused significant vegetative growth defects, abolished conidiation, and rendered the Δ*Modync1I2* strains non-pathogenic. Microscopic examinations revealed significant defects in microtubule network organization, nuclear positioning, and endocytosis Δ*Modync1I2* strains. MoDync1I2 is localized exclusively to microtubules during fungal developmental stages but co-localizes with the histone OsHis1 in plant nuclei upon infection. The exogenous expression of a histone gene, MoHis1, restored the homeostatic phenotypes of Δ*Modync1I2* strains but not pathogenicity. These findings could facilitate the development of dynein-directed remedies for managing the rice blast disease.

## Highlights

• DYNC1I2 plays moon-lighting roles in fungal morphogenesis and pathogenesis
• MoDYNC1I2 targets host nuclei during rice blast fungus-host interaction
• Histone1 partially rescued *MoDYNC1I2* deletion homeostatic defects in *M. oryzae*
• MoDYNC1I2 negatively regulates melanin biosynthesis in the rice blast fungus

## Graphical abstract

**Figure:**
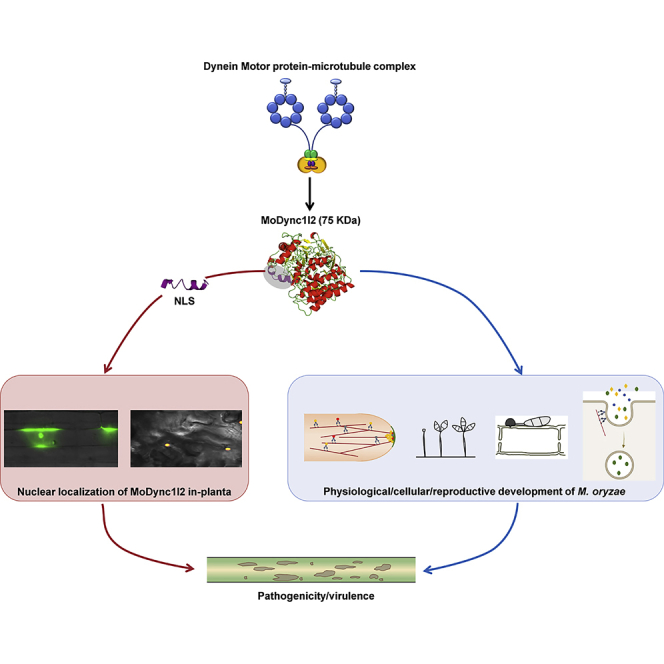

## Introduction

The principal factor threatening rice production worldwide is blast disease caused by the ascomycete fungus *Magnaporthe oryzae* B.Couch, which is additionally pathogenic on other cultivated grasses.^1^^,^^2^ The rice blast fungus is regarded as “one of the most important” or “an exceptionally important plant pathogenic fungus, given that rice provides the primary source of calories for over half of the world’s population.^3^ *M. oryzae* can infect all tissues of susceptible host plants; however, infection of the panicle, a highly branched flower cluster, can lead to complete loss of grain. Because losses of 10%-30% are typical for infected rice plants, regional epidemics can be devastating for crop yield.^3^

For successful infection, the rice blast fungus must undergo a series of conformational changes, in turn developing potent infectious structures including the germ tube, appressorium, and penetration peg.^4^ Also, studies showed that the commencement and progression of autophagic processes facilitate the efficient translocation of a variety of cellular molecules and organelles for successful pathologic differentiation of phytopathogenic fungi, including *M. oryzae*, during pathogen-host interaction and hence, positively regulate infection characteristics of the rice blast fungus.^5^ The successful transport of molecules and organelles within cells requires the action of motor proteins that operate in the context of the cytoskeletal system, such as microfilaments and microtubules.^6^^,^^7^^,^^8^

Cytoplasmic dynein belongs to a family of cellular motor proteins that move along microtubules by converting chemical energy stored as ATP into mechanical energy for movement.^9^^,^^10^ Cytoplasmic dynein facilitates the retrograde transport of cellular components including multivesicular bodies and other cargoes along microtubules toward the cell center or the minus-end of the microtubule.^11^^,^^12^ Structurally, cytoplasmic dynein is a dimer of dimers, containing two heavy chain globular protein “heads” with the ATPase activity required to generate the energy driving movement, plus various intermediate and light chains responsible for attaching the dynein complex to its cargo.^13^^,^^14^^,^^15^ Dynein-associated processes include endocytosis, retrograde transport of cargoes including organelles, and positioning of chromosomes and cell division structures during mitosis. The endocytic pathway is crucial for the growth, conidiation, and pathogenicity of the rice blast fungus *M. oryzae*.^16^^,^^17^ Additionally, retrograde transport is indispensable for the parasitic life of most pathogenic microbes by regulating the secretion of effector proteins during pathogen-host interactions.^18^^,^^19^

In this study, we aimed to identify additional factors contributing to the pathogenesis of *M. oryzae* by examining the predicted extracellulome of this pathogen. In the previous analysis, we identified cytoplasmic dynein 1 intermediate chain 2 (Dyn1I2) as a novel non-classically secreted protein in *M. oryzae* (data not shown here). Despite the well-established roles in dynein motor proteins in cellular growth and homeostasis, the direct or indirect contribution of these proteins to the morphological development and pathogenic differentiation of fungal pathogens, and to the progression of the host-pathogen interaction is unknown. In this study, we employed functional genetic, biochemical, and histopathologic studies to functionally characterize the contribution of *MoDYN1I2* to both the physiological and infectious development of rice blast fungus.

## Results

### Identification, phylogeny, and comparative structural analysis of *DYN1I2*

**Figure 1:**
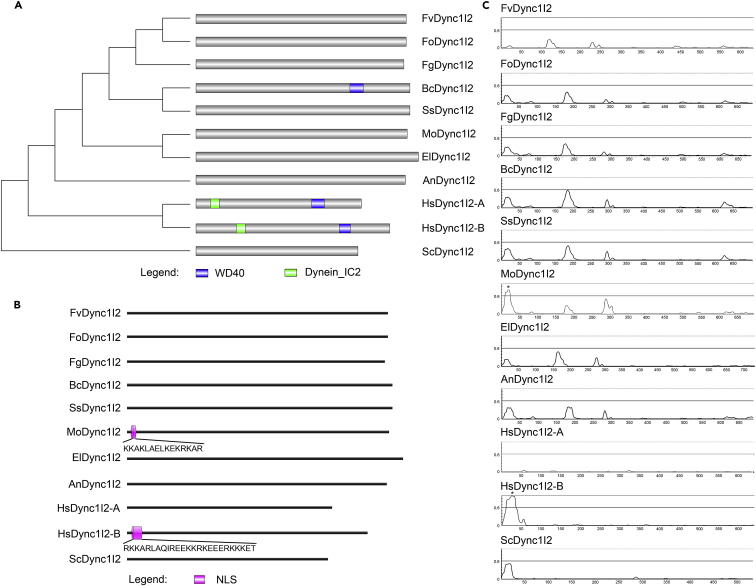
Phylogeny and domain structure of DYNC1I2 protein sequences identified in selected organisms (A) The likelihood neighbor-joining cladogram shows the phylogenetic relationship between MoDync1I2 and its corresponding orthologs in selected organisms, along with their predicted domain distribution and architecture. The tree was constructed with MEGA-X software and enhanced with the Interactive Tree of Life tool (iTOL; webserver/https://itol.embl.de/). (B) NLStradamus (http://www.moseslab.csb.utoronto.ca/NLStradamus/) based comparative analysis revealed the presence or absence of nuclear localization signal (NLS) peptide sequences in DYNC1I2 orthologs from the analyzed species. (C) Plots show the likelihood scores for NLS signals predicted in individual DYNC1I2 sequences in the selected organisms; sequences with NLS ≥0.6 are suggestive of the presence of NLS and denoted with a single asterisk (∗).

To identify cytoplasmic dynein 1 intermediate chain 2 (DYNC1I2) in *M. oryzae,* we retrieved the amino acid sequence of *Saccharomyces cerevisiae* dynein intermediate chain (YDR488C/PAC11) from the EnsemblFungi database (http://fungi.ensembl.org/Saccharomyces_cerevisiae/Info/Index), cross-referenced against the *S. cerevisiae* genome database (SGD; https://www.yeastgenome.org/) (Table S2). Results from BLASTp searches using the Fungal and Oomycete Informatics Resource database (https://fungidb.org/)^20^ revealed a single copy gene (MGG_04771/*MoDYNC1I2*) as the YDR488C ortholog present in *M. oryzae.* Further phylogenetic analysis showed that MoDyn1I2 shares close sequence homology with Dyn1I2 orthologs in filamentous fungal species, particularly *Eutypa lata*, the causal agent of Eutypa dieback disease of grapevine and other woody plants^21^^,^^22^ but is more distant from those in *S. cerevisiae* and *Homo sapiens* (Figures 1A and S1). Compared to Dync1I2 orthologs identified in *H. sapiens* (HsDync1I2-A and HsDync1I2-B) and *Botrytis cinerea* (BcDync1I2), those in all other tested species (*Fusarium verticillioides*/FvDync1I2, *Fusarium oxysporum/*FoDync1I2, *Fusarium graminearum*/FgDync1I2, *S. cerevisiae*/SsDync1I2, *M. oryzae*/MoDync1I2, *E. lata*/EiDyn1I2, *Aspergillus nidulans*/AnDyn1I2, *Sclerotinia sclerotiorum*/ScDyn1I2) lack the typical dynein and WD40 domains (Figure 1A). Additionally, of all Dync1I2 homologs identified in these species, MoDync1I2 and HsDync1I2-A, and HsDync1I2-B are unique in possessing an N-terminal nuclear localization signal (NLS)^23^ (Figures 1B and 1C). Together, these results suggested that MoDync1I2 may assume additional roles unique from Dync1I2 in most fungal species, with the presence of the NLS further implying that MoDync1I2 likely localizes to the nucleus in the rice blast fungus specifically.

### Localization of cytoplasmic dynein 1 intermediate chain 2 (DYNC1I2) in *M. oryzae*

**Figure 2:**
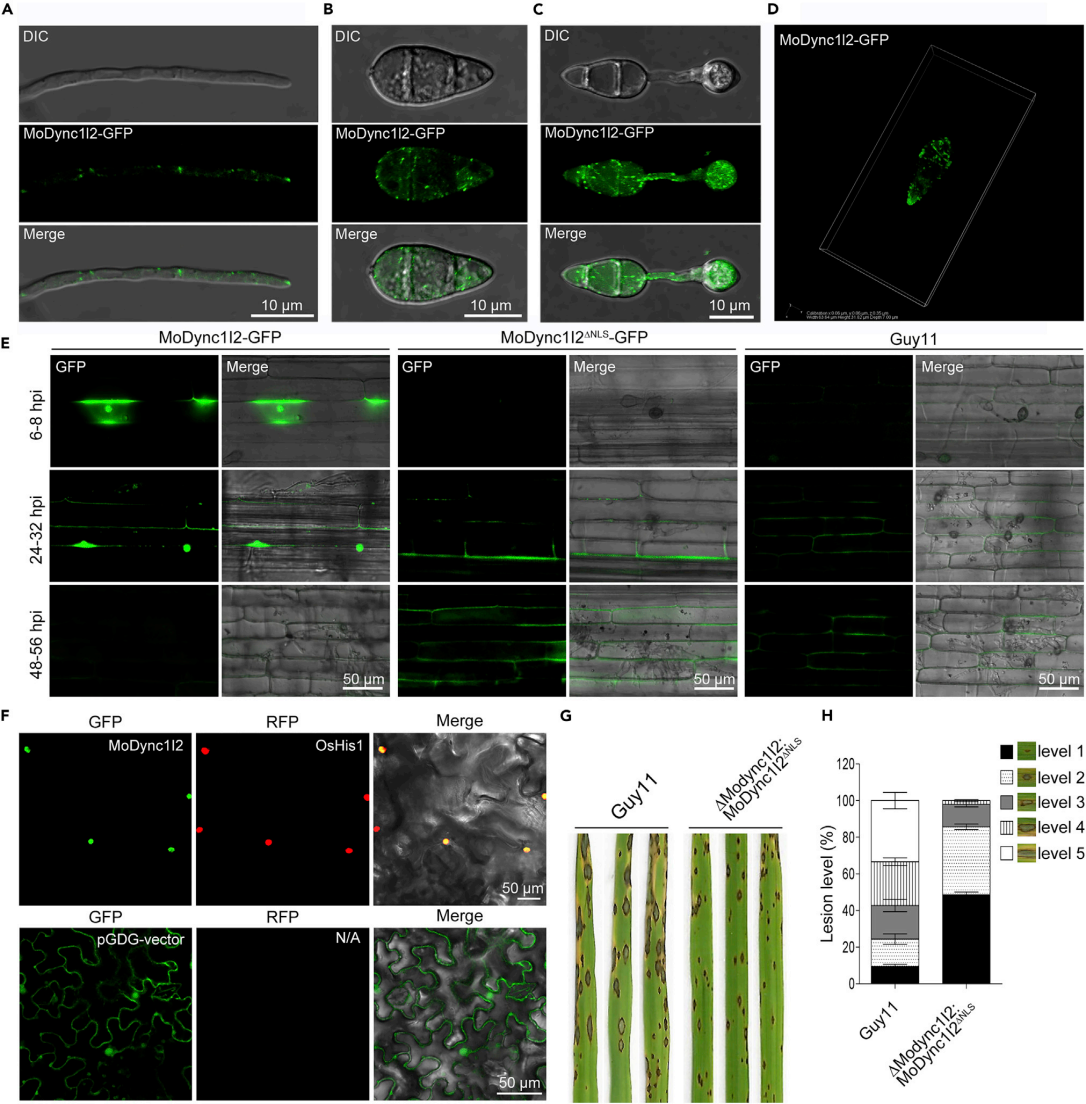
Dynamic localization of MoDync1I2 at different developmental and infectious stages of *M. oryzae* (A–C) Images show the subcellular localization pattern of MoDync1I2-GFP in the vegetative hyphae (A), in the asexual spores (B), and during the pathogenic differentiation of asexual spores (C), respectively. (D) A 3D microscopy image of MoDync1I2-GFP fluorescence in the conidia. (E) The histopathology micrograph of inoculated rice sheath tissues shows the secretion and accumulation of MoDync1I2-GFP fluorescence signal to the nucleus of inoculated rice sheath cells during the pathogen-host interaction in the early and mid-stages. (F) *Agrobacterium*-mediated transient transfection of *Nicotiana benthamiana* leaves with constructs encoding MoDync1I2-GFP and the rice histone 1 protein OsHis1-RFP confirmed the accumulation and co-localization of these proteins in the host cell nucleus. (G) Showed the virulence attributes of the MoDync1I2^ΔNLS^ against a three-week-old blast susceptible CO39 rice cultivar compared to the wild-type. (H) Statistical analyses of the different types (sizes) lesions induced on leaf tissues of three-week-old blast susceptible CO39 rice cultivar spray-inoculated with conidia suspensions prepared with asexual spores from the MoDync1I2^ΔNLS^ and wild-type strains. Note: For infection assays, seedlings were sprayed with 1 × 10^5^ conidia/mL in 0.02% Tween 20 suspension for each strain. The error bars represent standard errors from at least three independent replicates (∗∗, p < 0.01 by test). Micrograph scale bar = 20 μm. DIC indicates bright field illumination, GFP excitation was captured at 488 nm, and RFP was captured at 561 nm. Results in (F) represent three independent biological replicates, each consisting of three technical replicates.

To functionally assess the localization of this potentially unique dynein protein in *M. oryzae,* we transformed a MoDync1I2-GFP fusion construct into the Guy11 protoplast and subsequently monitored its dynamic localization during vegetative and pathogenic differentiation of the fungus. Microscopy examinations revealed the localization of MoDync1I2-GFP fluorescence to the cytoplasm and the plus-ends of microtubules in *M. oryzae* at all developmental stages (Figures 2A–2D and Video S1A–S1F). Given that our bioinformatic analyses identified MoDync1I2 as a non-classically secreted candidate effector protein that may target the host nucleus using its nuclear localization signal (NLS), we aimed to functionally test its potential translocation to the host nucleus upon infection. We inoculated leaf sheaths of a susceptible rice cultivar (LTH) with spore suspensions harvested from the MoDync1I2-GFP strain to monitor the potential secretion and localization pattern of MoDync1I2-GFP during early (12-20 hpi), mid (24-36 hpi), and late stages (48-72 hpi) of infection. Microscopic examination of the inoculated rice sheath tissues revealed the translocation and accumulation of MoDync1I2-GFP to the rice cell nucleus exclusively at the early and mid-stages of infection. However, it is nuclear why MoDync1I2-GFP was not detectable in the infected sheath cells at late stages (Figure 2E). Consistently, validation results using Agrobacterium-mediated transfection assays of *Nicotiana benthamiana* leaves further confirmed that MoDync1I2-GFP localized exclusively to the plant nucleus and co-localized with rice histone H1 (OsHis1) protein fused with RFP (OsHis1-RFP) co-infiltrated with MoDync1I2-GFP (Figure 2F). At the same time, we demonstrated that truncating the NLS located at close the N-terminal region of MoDync1I2, abolished the translocation of MoDync1I2^ΔNLS^-GFP to host nuclei (Figure 2E). Infections assessment results of Δ*Modync1I2:MoDYNC1I2*^ΔNLS^ revealed a substantial reduction in the virulence of the MoDync1I2^ΔNLS^ strains (Figures 2G and 2H). Based on these observations, we hypothesize that the nuclear translocation of MoDync1I2 may be involved in fungal pathogenesis. From these observations, we speculated that MoDync1I2 likely plays a moonlight role in promoting the physiological development of *M. oryzae* possibly by facilitating the polymerization of microtubule network on one hand, while on the other hand, functions as a putative non-classically secreted protein that likely targets and compromises the integrity of nuclear in the host cell during the progression of PHI.

Video S1. Subcellular localization pattern of MoDync1I2-GFP in the vegetative hyphae of *M. oryzae* (MERGE), related to Figure 2

### DYNC1I2 contributes to the vegetative development of the rice blast fungus

**Figure 3:**
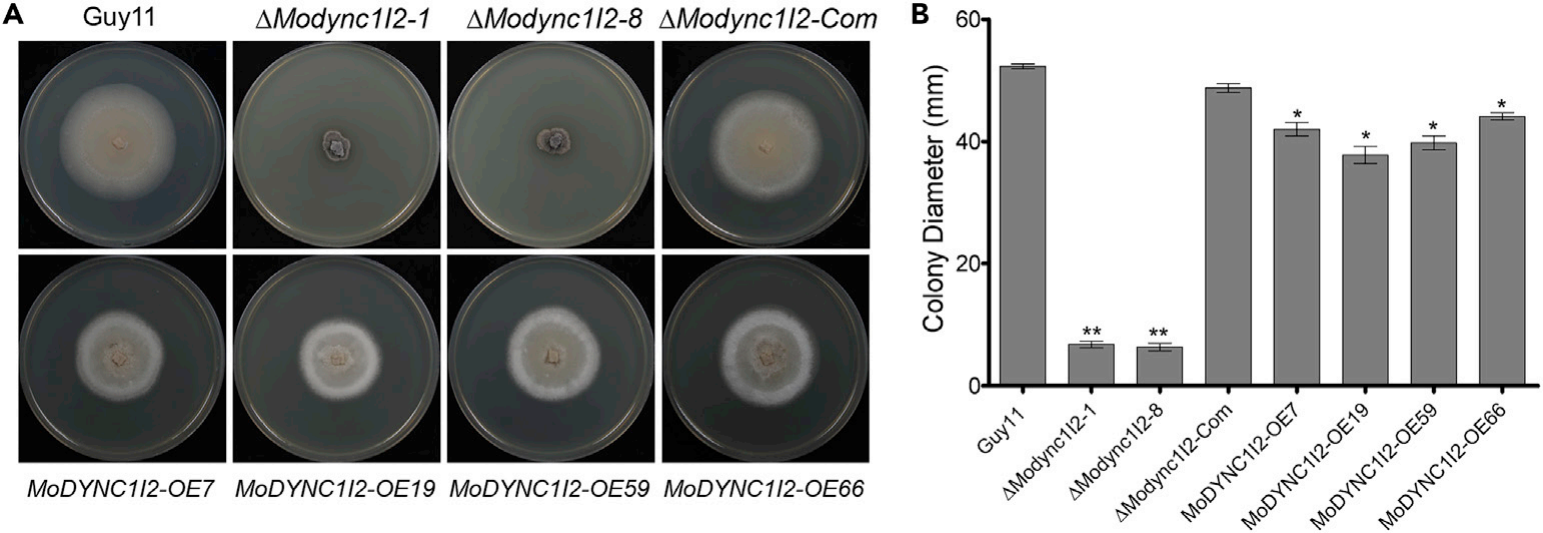
Impact of DYNC1I2 on the morphological development of rice blast fungus (A) Images depict the comparative vegetative growth performance of Δ*Modync1I2,* Δ*Modync1I*2*-Com, MoDYNC1I2-OE*, and the wild-type (Guy11) strain cultured on nutrient sufficient/complete media (CM) for 10 days. (B) Colony diameter measurements of each strain grown on CM for 10 days, from five independent biological experiments, each consisting of five technical replicates. One-way ANOVA(non-parametric) statistical analysis was carried out with GraphPad Prism8. Error bars show SD while single and double asterisks represent significance at ∗p < 0.05 and ∗∗p < 0.01.

To gain insights into the influence of MoDync1I2 on the morphological development of rice blast fungus, we generated *MoDYNC1I2* targeted gene deletion strains (Δ*Modync1I2)* using homologous recombination^24^ and overexpression (OE) strains by expressing MoDync1I2 under the RP27 constitutive promoter (Figure S2). Phenotypic characterization of Δ*Modync1I2* strains showed that targeted *MoDYNC1I2* deletion severely compromised vegetative development, accelerated melanization, and impaired radial differentiation of vegetative hyphae (Figures 3A and 3B, and Videos S1, S2, S3, S4, S5, and S6). Interestingly, the constitutive expression of MoDync1I2 similarly triggered notable, though less severe, impairment in the vegetative growth of the fungus (Figures 3A and 3B). Importantly, re-introducing the entire *MoDYNC1I2* ORF fused to GFP under the native promoter fully complemented the growth defects and abolished the hyper-melanization of the Δ*Modync1I2* strains (Δ*Modync1I2-Com*). These results suggest that MoDync1I2 directly or indirectly regulates cellular and physiological processes associated with vegetative differentiation and regulation of melanin biosynthesis in *M. oryzae.*

Video S2. Subcellular localization pattern of MoDync1I2-GFP in the vegetative hyphae of *M. oryzae* (GFP), related to Figure 2

Video S3. Subcellular localization pattern of MoDync1I2-GFP in the conidia of *M. oryzae* (MERGE), related to Figure 2

Video S4. Subcellular localization pattern of MoDync1I2-GFP in the conidia of *M. oryzae* (GFP), related to Figure 2

Video S5. Subcellular localization pattern of MoDync1I2-GFP during appressorium formation in *M. oryzae* (MERGE), related to Figure 2

Video S6. Subcellular localization pattern of MoDync1I2-GFP during appressorium formation in *M. oryzae* (GFP), related to Figure 2

### Dysfunction or hyperaccumulation of MoDync1I2 severely attenuates conidiogenesis in *M. oryzae*

**Figure 4:**
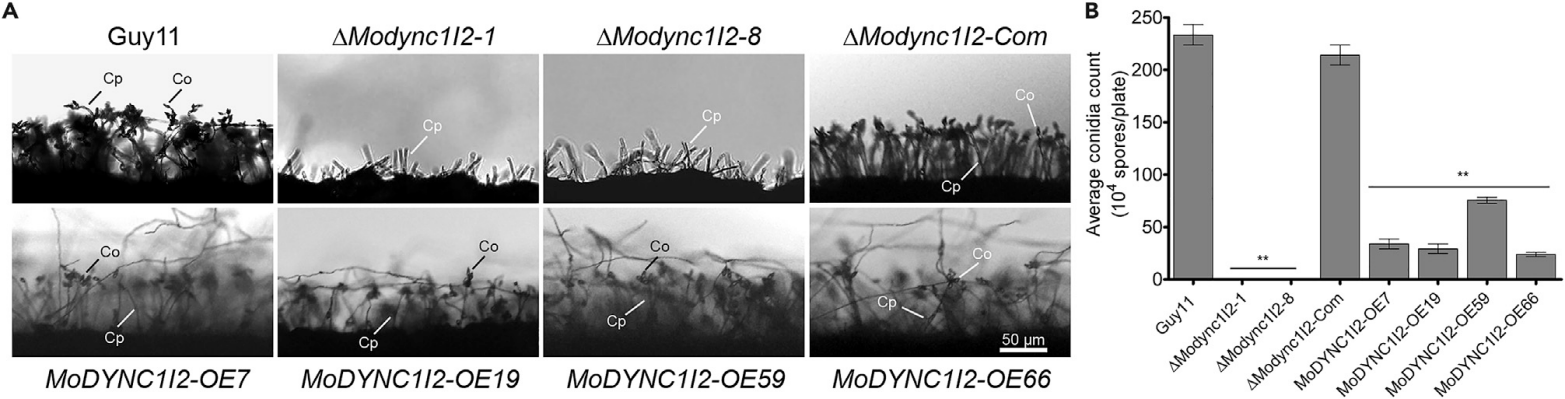
Changes in *MoDYNC1I2* dosage impair conidia production in *M. oryzae* (A) conidiophore imaging assays showed the impact of constitutive expression or targeted gene deletion of *MoDYNC1I2* on conidiophore (Cp) and conidia (Co) formation in *M. oryzae.* (B) The graph quantifies the conidiation of Δ*Modync1I2*, Δ*Modync1I2-Com*, *MoDYNC1I2-OE*, and wild-type strains grown on rice bran agar plates for 10 days. Data are from five independent biological experiments with five replicates each, and GraphPad Prism5 software was used for ordinary one-way ANOVA. The error bars denote SD∗p < 0.05 and ∗∗p < 0.001.

Asexual spores play vital roles in the dissemination of the rice blast disease. Therefore, to ascertain the contributions of MoDync1I2 to conidiogenesis in *M. oryzae.* We performed a comparative assessment of asexual reproduction characteristics of the genetically engineered strains and the wild-type by culturing the individual strains on conidiation-inducing rice bran agar (RBA) media for 10 days before scratching off the vegetative hyphae and exposing the plates to uninterrupted light (24 h) regimes to facilitate conidiophoregenesis and conidiation. Microscopic examination showed that targeted gene deletion of *MoDYNC1I2* caused a substantial reduction in conidiophore formation and completely abolished productive conidiation in the Δ*Modync1I2* strains (Figures 4A and 4B). Additionally, we observed that constitutive expression of MoDync1I2 also significantly suppressed conidiophore formation and conidia production (Figures 4A and 4B). These results suggest that either the absence or constitutive expression of MoDync1I2 exerts adverse effects on the progression of conidiogenesis in *M. oryzae*.

### MoDync1I2 influences both hyphae and spore-mediated initiation and establishment of rice blast disease

**Figure 5:**
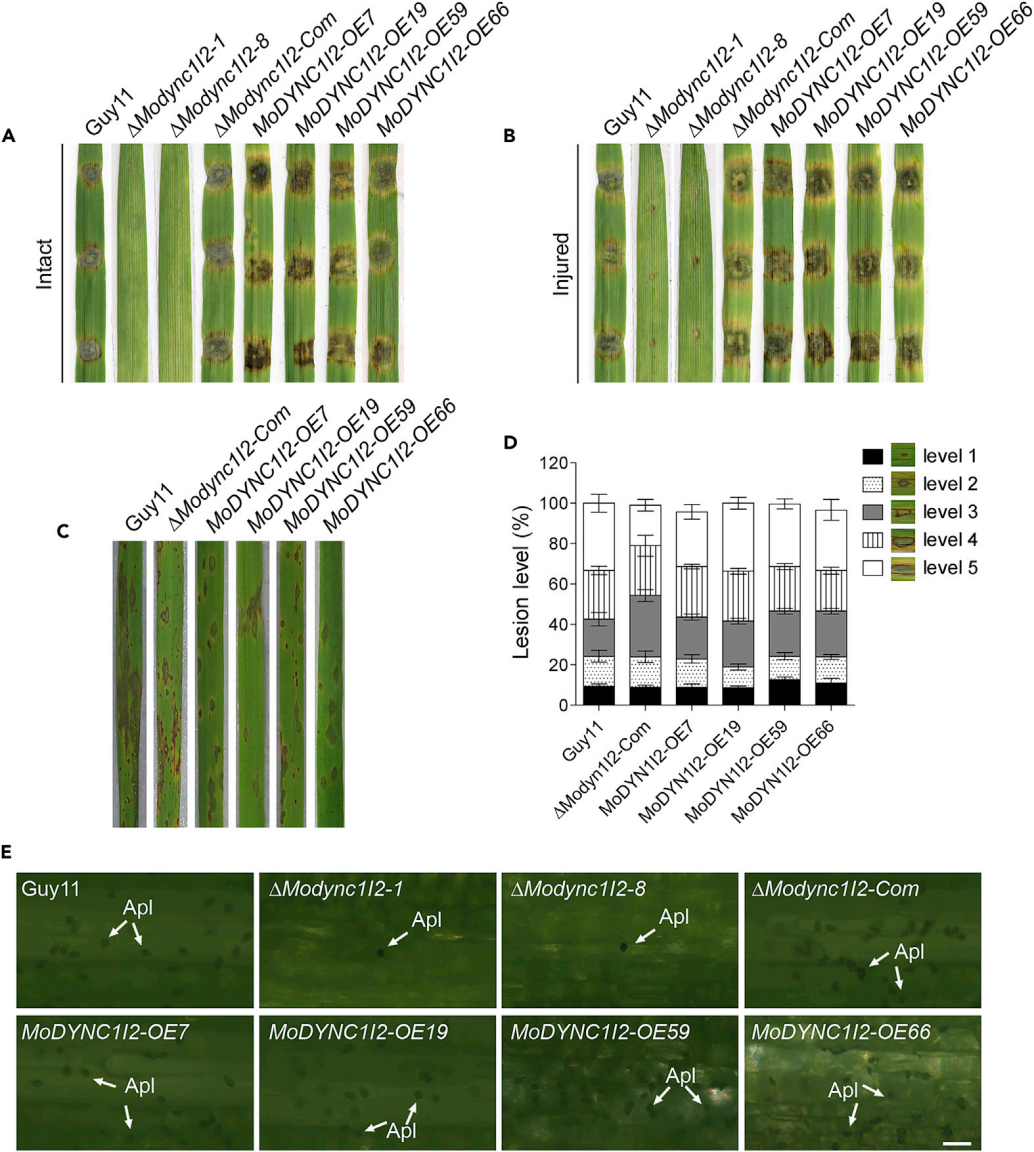
*MoDYNC1I2* deletion impairs the pathogenicity of *M. oryzae* (A) Images of infected leaves using hyphae-mediated infection of intact leaves of one-week-old Golden promise barley cultivar by Δ*Modync1I2*, Δ*Modync1I2-Com*, *MoDYNC1I2-OE*, and wild-type strains at 7-dpi. (B) Images of infected leaves using hyphae-mediated infection of injured leaves of one-week-old Golden promise barley cultivar by Δ*Modync1I2*, Δ*Modync1I2-Com*, *MoDYNC1I2-OE*, and wild-type strains at 7 dpi. Virulence impairment in Δ*Modync1I2* strains and enhancement in the virulence of the *MoDYNC1I2-OE* strains manifest in the induction of more prominent lesions on intact and injured leave compared to the wild-type. (C) Comparative virulence was assessed on three-week-old blast susceptible CO39 rice cultivar spray-inoculated with conidia suspensions prepared from the Δ*Modync1I2-Com*, *MoDYNC1I2-OE*, and wild-type strains. (D) Statistical analyses of the different types (sizes) lesions induced on leaf tissues of three-week-old blast susceptible CO39 rice cultivar spray-inoculated with conidia suspensions prepared with asexual spores from the Δ*Modync1I2-Com*, *MoDYNC1I2-OE*, and wild-type strains. Note: For infection assays, seedlings were sprayed with 1 × 10^5^ conidia/mL in 0.02% Tween 20 suspension for each strain. The error bars represent standard errors from at least three independent replicates (∗∗, p < 0.01 by test). (E) Display appressorium-like structures produced by the individual strains inoculated on barley leaves at 48-hpi. All infection results were generated with three biological experiments and five technical replicates, and for each strain, a total of five seedlings were inoculated under each technical replicate.

We showed that the deletion and overexpression of *MoDYNC1I2* negatively influenced the vegetative growth and conidiogenesis of *M. oryzae.* Next, to ascertain the direct or indirect impacts of *MoDYNC1I2* genetic manipulation on the pathogenicity and virulence of rice blast fungus, we performed hyphae-mediated infection assays by inoculating intact and injured leaves of blast susceptible rice (two-week-old CO39 cultivar) and barley (Golden promise cultivar) with mycelia harvested from Δ*Modync1I2,* Δ*Modync1I2-Com*, *MoDYNC1I2-OE*, and wild-type strains. We observed that the Δ*Modync1I2* strains failed to induce blast lesions on intact and injured rice and barley leaves (Figures 5A and 5B).

Concurrently we performed spray inoculation of blast susceptible rice and barley seedlings with 5 mL of spore suspensions (spore concentration = 1x10^5^/mL) prepared from asexual spores of the *MoDYNC1I2-OE*, Δ*Modync1I2-Com,* and wild-type strains, which confirmed that the constitutive expression of *MoDYNC1I2* substantially enhanced the virulence of the strains (Figures 5C and 5D). Furthermore, we monitored the formation of hyphae appressorium-like structures in the vegetative hyphae produced by the individual strains on barley leaves. Microscopy examinations showed that the targeted replacement of the *MoDYNC1I2* gene substantially suppressed the formation of appressorium-like structures in *M. oryzae* (Figure 5E). Together, these results suggest that the expression or function of MoDync1I2 requires tight regulatory control for optimal growth and pathogenesis of *M. oryzae.*

### Targeted deletion of *MoDYNC1I2* disrupts microtubule structure and nuclei positioning

**Figure 6:**
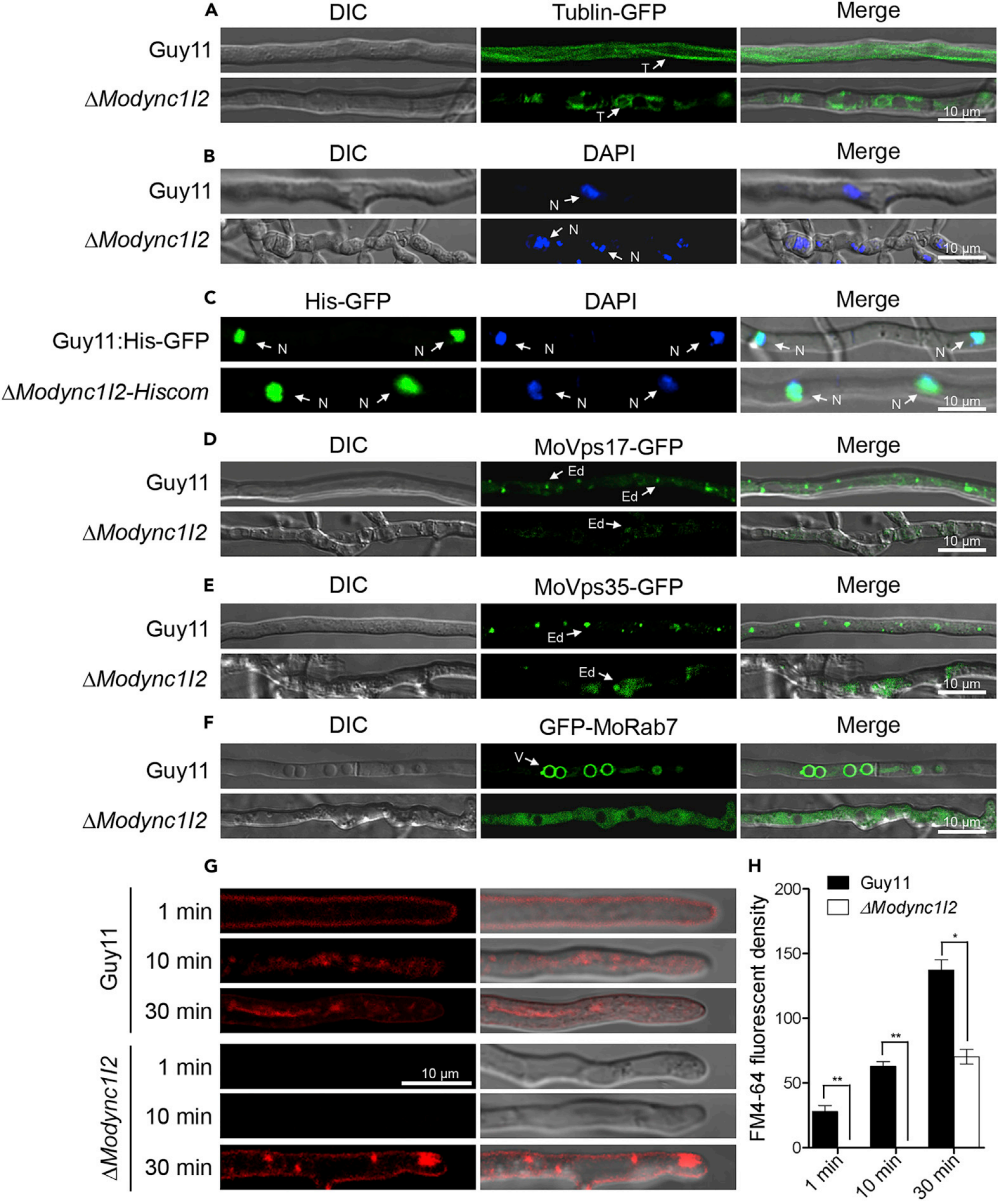
The influence of *MoDYNC1I2* gene deletion on microtubule polymerization, nucleation, retrograde trafficking, and endocytosis in the vegetative hyphae of *M. oryzae* (A) The organization of microtubule (T) architecture in the MoTubulin-GFP in the Δ*Modync1I2* strains compared to wild-type. (B) Δ*Modync1I2* and wild-type strains were stained with DAPI to visualize nuclear (N) morphology. (C) Comparative nuclear morphology of Δ*Modync1I2* and wild-type strains harboring histone 1 nuclear marker fused to GFP (His-GFP) and stained with 10 μg/mL of DAPI/nuclear-staining fluorescence dye is shown. (D–F) Comparative microscopic assessment of the progression of retrograde trafficking in Δ*Modync1I2* and wild-type strains using retrograde markers, including MoVps17, MoVps35 to by observing endosome (Ed) dynamics, and (F) MoRab7 fused to GFP for observing the progression of vacular (V) fusion. (G) The progression of endocytosis in the vegetative hyphae of the Δ*Modync1I2* and wild-type strains was tracked using 8 μM of the membrane-selective red fluorescent dye FM4-64, observed under a confocal microscope at 1, 10, and 30 min post-staining. (H) Showed results obtained from statistical computation of relative fluorescent density indexes. All scale bars = 20 μm. DIC indicates bright field illumination, blue fluorescence captured at 461 nm, and red fluorescence captured at 561 nm.

Cytoplasmic dynein motor proteins have historically been implicated in cytoskeletal polymerization, and depolymerization and nuclear positioning.^25^^,^^26^ Therefore, to evaluate the impact of *MoDYNC1I2* on microtubule structures, nucleus positioning, and related cellular processes, we transformed a Tubulin-GFP construct into Δ*Modync1I2* or wild-type strains to generate Δ*Modync1I2-*Tub and Guy11-Tub transgenic strains. Comparative microscopy examination of microtubule architecture in the Δ*Modync1I2-*Tub and Guy11-Tub strains revealed a severe disruption in the organization and polymerization of microtubules in the absence of *MoDYNC1I2* (Figure 6A). Furthermore, we stained vegetative hyphae produced by the Δ*Modync1I2* strains or wild-type with DAPI (4′,6-diamidino-2-phenylindole) and performed microscopy to assess the impact of *MoDYNC1I2* gene deletion on nuclear distribution. We also monitored the dynamic distribution of nuclei in the Δ*Modync1I2* and wild-type strains harboring a histone1-GFP fusion (MoHis-GFP) co-stained with DAPI using a confocal microscope. These experiments revealed that the deletion of *MoDYNC1I2* compromised nuclear migration, positioning, and morphology and resulted in the accumulation of multiple nuclei in single cells (Figure 6B). Interestingly, introducing MoHis-GFP into Δ*Modync1I2* restored nucleation defects and normalized nuclear distribution but failed to fully rescue abnormalities in nuclear morphology (Figure 6C).

Next, to assess the consequence of *MoDYNC1I2* absence on endocytic transport in the rice blast fungus, we assessed the localization of proteins associated with the endocytic transport pathway, including MoRab7 (GFP-MoRab7), MoVps17 (MoVps17_GFP), and MoVps35 (MoVps35_GFP). These results showed that the targeted gene replacement of *MoDYNC1I2* interfered with the localization of MoRab7_GFP, MoVps17_GFP, and MoVps35_GFP, indicating that MoDYNC1I2 plays a vital role in the progression of endocytic transport in *M. oryzae* (Figures 6D-6F). Staining with FM4-64, a dye that facilitates the microcopy assessment of the progression of the endocytosis process. Results from these examinations further confirmed impairment in the endocytic pathway upon *MoDYNC1I2* deletion (Figures 6G and 6H). We thus conclude that MoDYNC1I2 exerts a significant influence on the morphological and pathological development of rice blast fungus, likely through the regulation of diverse cellular processes, including endocytic transport, nuclear distribution, and organization of cellular structures.

### Histone 1 partially complements the growth, conidiation, and hyper-melanization defects associated with *MoDYNC1I2* deletion

**Figure 7:**
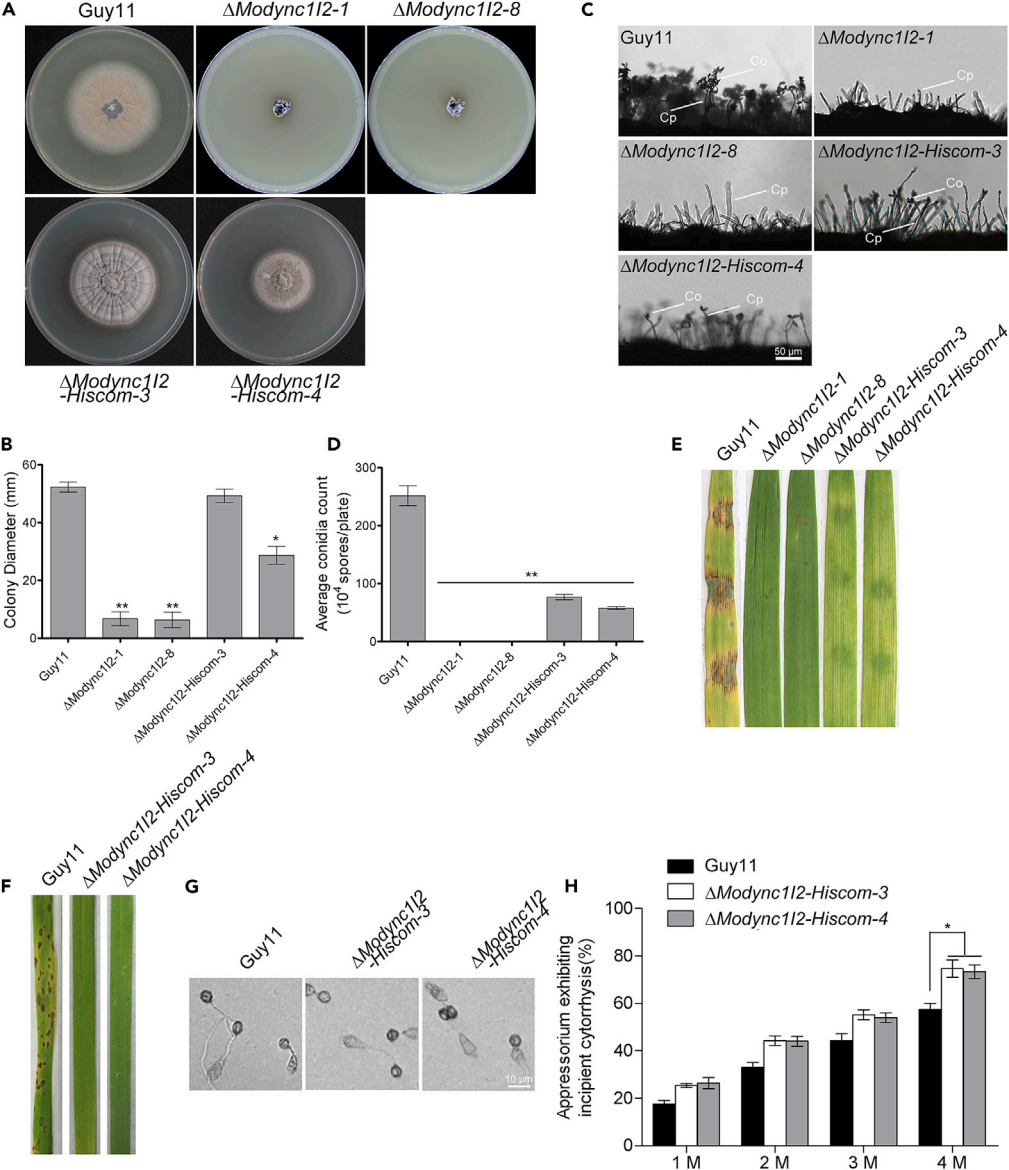
Histone 1 protein partially rescues vegetative growth and sporulation defects but does not restore the pathogenicity of Δ*Modyn1I2* strains (A) Δ*Modync1I2*, Δ*Modync1I2-Hiscom*, and wild-type strains were cultured on CM for 10 days. His1-GFP partially restored the vegetative growth of Δ*Modync1I2* strains (Δ*Modync1I2-Hiscom*). (B) The comparative colony diameter of Δ*Modync1I2*, Δ*Modync1I2-Hiscom*, and wild-type strains cultured on CM for 10 days, n = 5 biological replicates. (C) Microscopy imaging showed conidiophore (Cp) formed in the Δ*Modync1I2-Hiscom* strains bearing conidia (Co) as indicated with white arrows. While conidiophore (Cp) produced by the Δ*Modync1I2* strains failed to bear conidia. (D) The plots show the conidiation efficiency of the Δ*Modync1I2-Hiscom*, Δ*Modync1I2*, and wild-type strains cultured on rice bran agar media for 10 days. (E) Infection by Δ*Modync1I2-Hiscom*, Δ*Modync1I2*, and the wild-type strains on leaf tissues of one-week-old seedlings of the golden promise barley cultivar. (F) The failure of conidia suspensions prepared from conidia obtained from the Δ*Modync1I2-Hiscom* strains to cause blast lesions on leaf tissues of three-week-old blast susceptible CO39 rice cultivar spray-inoculated, seedlings were spray-inoculated with one × 10^5^ conidia/mL in 0.02% Tween 20 suspension. The experiment was replicated three times, and in each experimental set-up, 3-seedlings were inoculated with each strain; thus, the total number of seedlings (n) per strain is given as (n = 3∗3). (G) The micrograph represents the results from incipient cytorrhysis-based measurement appressorium turgor in the Δ*Modync1I2-Hiscom* strains treated with different concentrations (1, 2, 3, and 4 M) of glycerol compared to the wild-type. Appressorium formation was induced by inoculating conidia harvested from the individual strains on appressorium-inducing hydrophobic coverslips for 8 h prior to glycerol treatment. (H) Statistical computation of the number of collapsed appressorium observed and counted for the Δ*Modync1I2-Hiscom*, and the wild-types treated with different concentrations of glycerol treatment. For each biological replicate, 100 appressoria were counted (n = 100 × 3). Asterisks “∗” represent a statistically significant difference of p ≤ 0.05. The results were obtained from three independent biological experiments with three replicates each (number appressorium counted per each technical replicate = 100). The results in (B) and (D) represent five independent biological experiments with five replicates. The GraphPad Prism5 software was used for one-way ANOVA analyses. The error bars represent SD while single (∗) and double (∗∗) asterisks represent a p ≤ 0.05 and p ≤ 0.001, respectively.

While our initial intention was to monitor the impact of *MoDYNC1I2* deletion on nuclear positioning, we observed that the transformation of His1-GFP into Δ*Modync1I2* strains *(*Δ*Modync1I2-hiscom*) not only reversed the nucleation defects and nuclear distribution of Δ*Modync1I2* strains but also partially rescued their vegetative growth, and hyper-melanization defects (Figures 7A and 7B). In light of this observation, we proceeded to examine conidiation and pathogenesis in the Δ*Modync1I2-hiscom* strains. We found that while Δ*Modync1I2-hiscom* strains are now able to produce conidia (Figures 7C and 7D), they remain non-pathogenic against blast susceptible rice and barley seedlings (Figures 7E and 7F). These results indicate that histone overexpression is sufficient to restore a subset of phenotypes conferred by *MoDYNC1I2* deletion relating to growth and asexual development, but cannot restore the pathogenicity of the fungus, suggesting that the effects of MoDync1I2 on infective potential may be independent of these homeostatic functions.

### Relative expression of putative histone, melanin biosynthesis, conidiation, and kinesin motor protein genes

**Figure 8:**
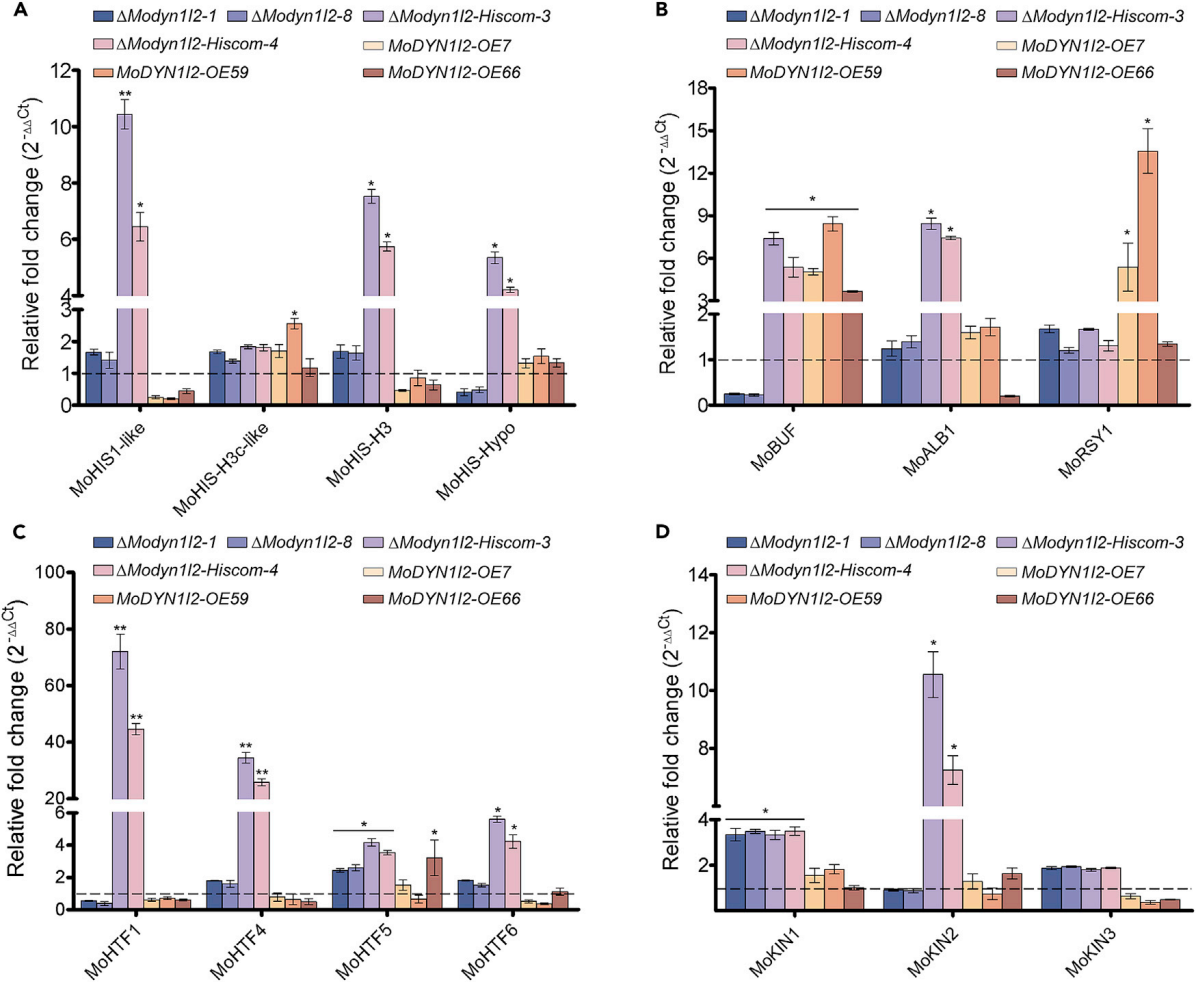
Comparative expression pattern of genes encoding proteins associated with anterograde transport, histones, pigmentation/melanin biosynthesis, and conidiation-related transcription factors in *ΔModync1I2* and *ΔModync1I2-Hiscom* strains (A–D) The relative expression of genes encoding histone proteins (A) pigmentation genes (B) conidiation-associated homeobox transcription factors (C) or anterograde transport machinery components (D) were measured during vegetative growth of *ΔModync1I2*, *ΔModync1I2-Hiscom*, *MoDYNC1I2-OE* and wild-type strains by RT-qPCR. All results are from three biological replicates, with each consisting of three technical replicates. All results are from three biological replicates, each consisting of three technical replicates. For all panels, the gene encoding actin (*MoACTIN*) expression was used as the reference gene, the expression of each gene in the wild-type strain was used as the internal control, and the delta delta-CT method (2^−ΔΔCT^) was used for data analysis. The error bars represent SD while single (∗) and double (∗∗) asterisk represent a p ≤ 0.05 and p ≤ 0.001, respectively.

We used a targeted gene expression analysis to determine the consequences of histone 1 complementation in the Δ*Modync1I2* mutant. Because histone-1 protein transformed into Δ*Modync1I2* (Δ*Modync1I2-hiscom*) partially complemented its growth and conidiation defects, we decided to examine the relative expression levels of putative histone proteins in these strains. Results showed that the expression levels of *MoHis1-like, MoHis-H3c-like*, and *MoHis-H3* are lower in the Δ*Modync1I2* strains compared to Δ*Modync1I2-Hiscom*. the expression of *MoHis1-like* and *MoHis-H3* increased approximately 6-fold in the Δ*Modync1I2-Hiscom* strains. In comparison with wild-type, expression of *MoHis-Hypo* decreased significantly in the Δ*Modync1I2* strains while it increased ∼5-fold in the Δ*Modyn1I2-Hiscom* strains. Overall, we found that introducing His1-GFP constructs into Δ*Modync1I2* strains enhanced the expression *MoHis1-like*, *MoHis-H3c-like, MoHis-H3*, and *MoHis-Hypo* in the Δ*Modync1I2* strains. At the same time, we observed that the overexpression of MoDync1I2 enhanced the expression pattern of *MoHis1-like*, *MoHis-H3c-like*, and *MoHis-H3* (Figure 8A).

Next, given the enhancement in the melanization of vegetative hyphae produced by the Δ*Modync1I2* strains, we employed RT-qPCR assays to evaluate the expression of melanin and other pigmentation-associated genes, including tetrahydroxynaphthalene (*MoBUF1*/MGG_02252), polyketide synthase (*MoALB1*/MGG_02252), and rosy (*MoRSY*/MGG_07219). These results revealed a significant upregulation in the expression of *MoRSY1* in all Δ*Modync1I2* strains and suppression of the expression of *MoBUF1*. The latter effect was reversed by His1 addition, with *Modync1I2-hiscom* strains showing significant increases in both *MoBUf1* and *MoALB1* transcripts. At the same time, we observed that MoDync1I2 overexpression equally triggered significant upregulations in the expression of *MoBUF1* and *MoRSY1* (Figure 8B). These results suggested a remodeling of gene expression relating to melanization upon transfection with His1.

Homeobox transcription factors (HTFs) have been implicated in the conidiogenesis of multiple filamentous fungal pathogens, including *M. oryzae*.^27^^,^^28^ Thus, we investigated the impact of *MoDYNC1I2* gene deletion and MoHis1 transduction on the expression levels of conidiation-associated HTFs in rice blast fungus using RT-qPCR. We found a significant enhancement in the expression levels of both *MoHTF1* and *MoHTF4* in the Δ*Modync1I2-hiscom* strains relative to Δ*Modync1I2*, but no change in *MoHTF5* or *MoHTF6* levels. The overexpression of MoDync1I2 did not alter the expression pattern of the HTFs investigated in this assay (Figure 8C).

Finally, we evaluated the influence of *MoDYNC1I2* gene deletion on the transcription of kinesin motor protein genes in *M. oryzae*, as dynein and kinesin are known to regulate each other’s trafficking activities. We assessed *MoKIN1, MoKIN2*, and *MoKIN3* gene expression profiles by RT-qPCR. Results showed that the expression levels of *MoKIN1* and *MoKIN3* increased significantly (∼3-fold and ∼ 1-fold, respectively) in both Δ*Modync1I2* and Δ*Modync1I2-hiscom* strains compared to wild-type (Figure 8D). Also, while *MoKIN2* expression in Δ*Modync1I2* strains is indistinguishable from that of wild-type, there is a highly significant upregulation in its expression in the Δ*Modync1I2-Hiscom* strains. Contrary to the expression pattern recorded in the Δ*Modync1I2*, we observed that the kinesin coding genes displayed minimal expression patterns in the MoDync1I2 overexpression strains (Figure 8D). Together, these transcriptomic results show broad alterations in the expression of genes involved in nucleosome organization, conidiation, melanization, and anterograde transport (kinesin) in strains lacking *MoDYNC1I2*, many of which are reversed by the addition of His1.

## Discussion

Exocytic processes, including the trafficking of vesicles, proteins, and other cargoes, crucially regulate microbial pathogens' physiological and pathological development.^29^^,^^30^ Minus-end-directed transport processes in terrestrial plants, including rice, are performed by kinesin-14s due to the absence of cytoplasmic dynein in contrast to animals and fungi,^31^ the comparative impact of this imbalance in retrograde transport mediators pathogen-host interaction is still unknown. In the rice blast fungus, we identified a novel cytoplasmic dynein 1 intermediate chain 2 (DYNC1I2) as a possible secreted effector protein. However, further downstream studies are needed to validate the effector role of MoDync1I2 in *M. oryzae*. Subsequently, we demonstrated that although MoDync1I2 shared a high protein sequence identity and homology with orthologs identified in other fungal species and humans, all fungal DYNC1I2 proteins lack the typical dynein_IC2 domain present in the human protein. In contrast to other fungal orthologs but similar to the human protein, MoDync1I2 possesses an NLS at its N terminus. These findings suggested that fungal DYNC1I2 structurally diverged from their human counterparts in the early stages of evolution and nominated MoDync1I2 as an atypical dynein motor protein with diverse functions.

Dyneins are traditionally microtubule-associated motor proteins that mediate a plethora of cellular processes, including intracellular vesicle trafficking, positioning of organelles, and cell division.^32^^,^^33^ Although cytoplasmic dyneins preferably localize to cytoskeletal components, particularly microtubule polarization sites, their localization has been shown to be dynamic in different cell types depending on the physiological or developmental stage.^34^^,^^35^^,^^36^ Studies have shown that during the onset of cell division, dyneins preferably localize to the nuclear envelope in human and Drosophila cells through the action of Asunder.^37^

Analysis of the amino acid sequence of MoDync1I2 revealed the presence of an NLS; however, similar to the localization pattern reported for dynein in other filamentous fungi, including *A. nidulans*,^38^ we observed that MoDync1I2-GFP displayed sustained cytoplasmic localization patterns along the microtubule during spore germination and appressorium formation and vegetative development of the rice blast fungus. Interestingly, histopathological examination of MoDync1I2-GFP during invasive growth of *M. oryzae* revealed its accumulation in the host nucleus. Additionally, we showed that the deletion of the NSL region abolished the translocation of MoDync1I2 to the rice nuclei and reduced the virulence of MoDync1I2^ΔNLS^ strains. These observations suggested that MoDync1I2 assumes a role as a cytoplasmic effector protein during the development of rice blast fungus *in-planta*. Due to the absence of a canonical secretion signal peptide in MoDync1I22, we speculate that MoDync1I2 is likely secreted via an alternative secretion system.^39^^,^^40^^,^^41^

Cytoplasmic dyneins exert direct and indirect regulatory influences on multiple cellular and physiological events.^42^ Cellular morphogenesis is an energy-intensive process that requires the active trafficking of essential growth factors, including proteins, signaling peptides, and other cargoes, to and from sites of active cell division.^43^ In addition, efficient intracellular trafficking helps drive morphologic development by facilitating the recycling and transport of nutrients from aged cells or tissues to support new cell or tissue growth.^44^ Here, we found that targeted gene deletion of *MoDYNC1I2* triggered severe physiological defects, including a significant reduction in vegetative growth and attenuation in radial and polarized development of vegetative hyphae. At the same time, we showed that the constitutive expression of *MoDYNC1I2* also caused a substantial reduction in the vegetative growth of *M. oryzae*. These observations, coupled with previous studies implicating dynein motor proteins in the morphological development of *Drosophila melanogaster* and multiple fungal species, including *Candida albicans, Neurospora crassa,* and *A. nidulans*,^34^^,^^38^^,^^45^^,^^46^ supported our hypothesis that *MoDYNC1I2* plays a pleiotropic conserved role in the morphological development of diverse organisms. Furthermore, the impairment of morphological differentiation of vegetative hyphae caused by constitutive expression of *MoDYNC1I2* suggests the intracellular level of *MoDYN1I2* is likely under tight internal regulation in *M. oryzae*.

Motor protein-driven processes, including endocytosis, autophagic clearance, endosomal signaling, vesiculation, and organelle transport and positioning, have been linked directly or indirectly not only to morphogenesis but also to a wide array of physiological transformations in filamentous fungi, including reproduction, stress tolerance and pathogenesis.^47^ Our study showed that MoDync1I2 dysfunction disorganized the microtubule structure (MTS), impaired endocytosis and retrograde transport, abolished asexual reproduction, and rendered the Δ*Modync1I2* strains non-pathogenic on susceptible barley and rice seedlings. Since the efficient mobilization and trafficking of secretory vesicles, proteins, and nutrients, including growth and virulence factors, requires the coordinated actions of the entire cytoskeleton and associated motor proteins, we inferred that the pleiotropic reproduction and pathogenesis defects displayed by the Δ*Modync1I2* strains could be attributed to the fact that the deletion of *MoDYNC1I2* subverts coordination within the intracellular transport machinery. Although we have yet to examine the influence of *MoDYNC1I2* deletion on the initiation of autophagic processes in rice blast fungus, the role of cellular transport in autophagy-mediated nutrient recycling is crucial.^48^

During cell cycle progression in eukaryotes, the intrinsic localization of centrosomes in the nuclear envelope is orchestrated by microtubules under the influence of dynein motor proteins.^49^^,^^50^ Previous studies showed that dyneins act in association with dynactin via the microtubule-spindle pole network to partly mediate the disappearance of nuclear membranes by pulling the spindle poles toward opposite ends of the cell.^51^^,^^52^ We demonstrated that targeted gene disruption of *MoDYNC1I2* in the rice blast fungus triggered abnormal nuclear distribution. In *C. albicans*, targeted gene deletion of cytoplasmic dynein impaired the transfer of daughter nuclei to the emerging daughter cells prior to cytokinesis.^45^ Similarly, the deletion of cytoplasmic dynein heavy chain suppressed nuclear distribution and severely compromised cell cycle progression in *N. crassa* and other filamentous fungi.^38^^,^^46^^,^^47^ Thus, our results are consistent with prior work implicating cytoplasmic dynein in nuclear homeostasis in fungi.

Interestingly, the constitutive expression of kinetochore histone-like protein 1 (His1) partially rescued the morphological and sporulation defects associated with the Δ*Modync1I2* strains. Generally, cytoplasmic intermediate chain dyneins facilitate the targeting or linkage between enzyme complexes and other cellular components, including membrane-bound organelles, kinetochores, and various cargoes.^34^ During mitosis, the localization of dynein switches from vesicular to locazation to the spindle and kinetochores.^53^ Accordingly, we hypothesized that MoDync1I2 positively regulates morphological and asexual sporogenesis in *M. oryzae* by modulating the formation of MT-spindle or MT-kinetochore contacts to drive efficient cell cycle progression and proper nuclear segregation. therefore, the targeted gene replacement of *MoDYNC1I2* possibly suppressed the expression of histone by limiting MT-kinetochore contacts or attenuated histone balance in the *MoDYNC1I2* defective strains. Of note, constitutive expression of MoHis1 does not rescue the pathogenicity defects associated with Δ*Modync1I2,* indicating that disruptions in the formation of the dynein-mediated microtubule-spindle network are likely not the factor responsible for compromised pathogenicity in the Δ*Modync1I2* strains. Furthermore, we speculated the likely existence of MoDync1I2 independent role in the pathogenesis of *M. oryzae*.

Finally, cytoplasmic dyneins and cytoplasmic kinesins are two opposing forces transporting cargoes along microtubules toward the minus end and the plus end, respectively.^54^^,^^55^^,^^56^ Here, we observed that targeted gene deletion of *MoDYNC1I2* triggered significant upregulation of *MoKIN1* and *MoKIN3* expression but suppressed the expression of *MoKIN2*. However, constitutive expression of MoHis1 resulted in a significant upregulation of *MoKIN2* in the *ΔModyn1I2-Hiscom* strains. These results suggested that *MoDYNC1I2* deletion upset the symmetrical balance of the cytoskeleton (cellular symmetry),^57^ which may account for the pleiotropic pathophysiological defects associated with the *ΔModync1I2* strains and their partial rescue by histone overexpression.

Taken together, our results have demonstrated that MoDync1I2 is a dynein protein that localizes to microtubules during vegetative growth and developmental stages of *M. oryzae* and is necessary for normal vegetative growth, conidiogenesis, and pathological development of this filamentous fungi. Moreover, our results highlighted the critical role of MoDync1I2 in intracellular transport, which could be partially compensated for by overexpression of the model chromatin component, histone 1*.* These findings will be valuable in facilitating the development of effective remedies for managing rice blast disease.

### Limitations of the study

The limitation of this study relates to phenotypes observed in the *MoDYNC1I2* defective strains compared to the Guy11 wild-type strain. Also, this study this referred to MoDync1I2 as probable non-classically secreted protein based on results obtained from prediction analyses, and in reference to orthologs in other species including Humans.

## STAR★Methods

### Key resources table

**Table:** 

REAGENT or RESOURCE	SOURCE	IDENTIFIER
**Bacterial and virus strains**		

*Agrobacterium* GV3101	Biomed	Cat# BC304-01

**Chemicals, peptides, and recombinant proteins**		

DAPI	Sigma-Aldrich	Cat# D9542
FM4-64	Thermo Fisher Scientific	Cat# F34653

**Experimental models: Organisms/strains**		

*Magnaporthe oryzae* Δ*Modync1I2* strains	This paper	N/A
*Magnaporthe oryzae* Δ*Modync1I2_Com* strains	This paper	N/A
*Magnaporthe oryzae* Δ*Modync1I2:*MoDync1I2^ΔNLS^-GFP	This paper	N/A
*Magnaporthe oryzae* Δ*Modync1I2_Hiscom* strains	This paper	N/A
*Magnaporthe oryzae MoDYNC1I2-OE* strains	This paper	N/A
*Magnaporthe oryzae* Δ*Modync1I2:*MoVps17-GFP	This paper	N/A
*Magnaporthe oryzae* Δ*Modync1I2:*MoVps35-GFP	This paper	N/A
*Magnaporthe oryzae* Δ*Modync1I2*:GFP*-*MoRab7	This paper	N/A
*Magnaporthe oryzae* Δ*Modync1I2:*Tublin-GFP	This paper	N/A
*Agrobacterium* MoDync1I2-GFP	This paper	N/A
*Agrobacterium* OsHis1-RFP	This paper	N/A
*Agrobacterium* pGDG	Michael M. Goodin et al.^58^	N/A

**Oligonucleotides**		

Primer for plasmid construction, see Table S1	This paper	N/A

**Software and algorithms**		

DNAMANx	LynnonBiosoft	https://dnaman.software.informer.com/
ImageJ	National Institute of Health	RRID:SCR_003070; https://imagej.net/
Graphpad Prism 7.0	GraphPad Software	https://www.graphpad.com/
MEGA7	MEGA Software	http://www.megasoftware.net/download_form

### Resource availability

#### Lead contact

Further information and requests for resources and reagents should be directed to and will be fulfilled by the lead contact, Justice Norvienyeku (jk_norvienyeku@hainanu.edu.cn).

#### Materials availability

Plasmids and other reagents generated in this study are available without restriction by requesting to lead contact, Justice Norvienyeku (jk_norvienyeku@hainanu.edu.cn).

### Experimental model and subject details

#### Strains and growth condition

The *M. oryzae* strain Guy11 was provided by Dr. Didier Tharreau of PHIM, CIRAD, INRAE, IRD, Montpellier SupAgro, MUSE, Montpellier Cedex 05, France. The Guy11 strain was used as the background (wild-type) strain for generating targeted gene deletion, complementation, and transgenic strains. The genetically manipulated strains, including Δ*Modync1I2-1,* Δ*Modync1I2-8, ΔModync1I2_Com, ΔModync1I2:*MoDync1I2^ΔNLS^-GFP, *ΔModync1I2_Hiscom*, *MoDYNC1I2-OE*, *ΔModync1I2:*MoVps17-GFP*, ΔModync1I2:*MoVps35-GFP, *ΔModync1I2:*GFP-MoRab7, and the *ΔModync1I2:*Tublin-GFP were constructed in this study.Protoplast preparation, RNA/DNA extraction, and vegetative growth analysis was performed by culturing the strains liquid complete media (CM, 6 g yeast extract, 6 g casein hydrolysate, 10 g sucrose, 20 g) under the dark condition under stable temperature of 28°Cfor for 3-days, The strains were cultured on rice bran media (RBM, 40 g rice bran, 20 g agar, and pH 6) for conidiation assessment assays.

### Method details

#### Generation of deletion mutants and complementation

Targeted gene deletion using a homologous recombination strategy was used to generate the Δ*Modync1I2* mutants. Gene deletion constructs were obtained by fusion PCR of ∼1 Kb upstream of the ORF, the hygromycin resistance gene with ∼1 Kb downstream of the ORF, cloned into pCX62.^59^ The split marker constructs were obtained by cloning of ∼1 Kb regions downstream and upstream flanking sequences of *MoDYNC1I2* into the plasmids using the primer HY and YG respectively. HY was obtained by cloning the 5’ end of the hygromycin phosphotransferase gene (hph) with primer pairs HYG-F/HY-R and YG by cloning the 3’ end with primers YG-F/HYG-R.^60^ For complementation, we first amplified the upstream fragment of the *MoDYN1I2* sequence containing the native promoter with the complete ORF lacking the stop codon from the wild-type Guy11 strain gDNA. Next, we purified the resultant PCR product and cloned it into the *Kpn* I /*Hin*d III site of the plasmid pKNTG upstream of C-terminal GFP using ligation independent cloning (LIC). The resulting complementation construct was sequenced to validate the orientation of the inserted fragment, and then transformed into Δ*Modync1I2* deletion mutant protoplasts through PEG-mediated transformation.

#### Assays for vegetative growth, conidiation and pathogenicity

For the vegetative growth assay, wild type Guy11, mutants, and complemented strains were grown on CM media for five days. Mycelia plugs were placed onto the freshly prepared CM agar on 90 mm plates and cultured in the dark at 28°C. The diameter of fungal colonies was measured 10 days post incubation. For the conidiation assay, the mycelial plugs were cultured on RBM and incubated for 10 days in the dark. The hyphae were carefully scratched using sterilized glass slides and the plates were kept in an incubator at 28°C under light for 3 days. Thereafter, the conidia were harvested by washing the fungal mycelia on the culture plates using sterilized ddH_2_O. The conidia suspension was then filtered through one layer of lens cleaning paper, and volume adjusted to 2 mL, then counted using a haemocytometer.

The pathogenicity assay was carried out in two different ways. For the first pathogenicity assay, mycelia were used to infect the detached rice and barley leaves as the Δ*Modync1I2* mutants don’t produce conidia. Mycelial plugs of similar sizes (2 mm diameter) from the Guy11 and mutant strains (5 days old on CM solid media) were cultured in liquid CM with gentle shaking at 150 rpm for 2 days at 28°C. The mycelia were harvested using a funnel and single layer Whatman filter paper followed by washing with distilled water twice, and dried on Whatman filter paper. Wounded leaves were prepared by removing the surface cuticle by abrasion with pipette tip. The hyphae were inoculated onto the detached leaf surface of intact or injured leaves. The inoculated plants were incubated in the dark on a plastic plate with full humidity at 25°C for 24 hours. The disease lesions were examined and photographed at 7 days post inoculation.

For the second pathogenicity assay, wild type Guy11, Δ*Modync1I2-com* and Δ*Modync1I2-Hiscom* mutant strains were grown on RBM media and conidia suspensions were harvested as described above. The conidia suspension was adjusted to a concentration of 5×10^4^ spores per milliliter, and 5 mL of 5x10^4^ /mL were sprayed on 2-week-old rice seedlings. Prior to spraying, Tween 20 was added as described by^61^ to promote the adherence of conidia to the rice leaf surface. The inoculated plants were incubated in a dark chamber at 25°C with 90% humidity for 24 hours as high humidity is required for fungal penetration into the host plant cuticle, followed by a 12/12 hour light/dark cycle. Disease lesions were monitored daily and photographed 7 days after inoculation.

#### RNA extraction and quantitative real time PCR (qRT-PCR) analysis

Total RNA was extracted from mycelia, which were grown in liquid complete medium using universal RNA kit (Magen Biotech) according to the manufacturer’s protocol. The cDNA was synthesized using a two-step Vazyme RT-PCR kit according to manufacturer’s protocol. qPCR reactions were performed with primer pairs listed in (Table S2).

#### Phylogenetic analysis

The amino acid sequences were obtained from the NCBI database (http://www.ncbi.nlm.nih.gov) by blasting the protein sequence of *MoDYNC1I2.* The identified homologs in *M. oryzae* were confirmed by BlastP search in the Fungi and Oomycetes genomics resources database (http://fungidb.org/fungidb/). Analysis of the domain architecture was performed with pfam software (www.pfam.xfam.org/). Multiple protein sequence alignments were performed using DNAMAN software while phylogenetic analysis was performed with MEGA7 software and evolutionary relationships inferred using the Maximum Likelihood method at bootstrap 1000.

#### In planta localization of MoDync1I2 and agrobacterium mediated transient expression

The transfection assay was carried out with MoDync1I2-GFP and rice histone1 (OsHis1-RFP) according to the procedure described in.^58^ *Agrobacterium tumefaciens* strain GV3101 competent cells were transformed with pGDG containing our gene of interest (*MoDYNC1I2*). Blunt end syringe was used to perform the infiltration on 4-week-old tobacco leaves. The infiltrated tobacco plants were kept in the dark at 48°C for 3 days. The fluorescent proteins were observed at 3 dpi under a laser scanning confocal microscope.

#### Light microscopy and confocal microscopy assay

Transmission electron microscopy was performed as described previously by.^62^ We checked for conidia and conidiophore under the Nikon TiE system (Nikon, Japan). The microtubule network, nuclear positioning and internalization of FM4-64 were monitored using a confocal microscope at a wavelength of 561 nm. Green and Red fluorescent were monitored using standard light microscopy at 488 nm, and 561 nm, respectively.

### Quantification and statistical analysis

Statistical analyses were performed using Prism 5 (GraphPad Software) or Microsoft Excel (Microsoft Corporation). The two-way non-parametric ANOVA (Friedman ANOVA Test) was used to assess differences. All the experiments were replicated at least three times as stated in the corresponding figure legends. Quantification of fluorescence signals in localization/co-localization assays were performed using the Image-ProPlus 6.0 software. The bi-directional bar indicated SEM, The “n”, used in the figures and figure legends, denote the total number of experimental samples examined in at least three biological replicates, as indicated in the relevant figure legends. p ≤ 0.01 or p ≤ 0.05 was used as a measure of statistical significance and statistical analyses with p-value of p ≤ 0.01 or p ≤ 0.05 are represented as double (∗∗) or a single (∗) asteriks in the figures constructed with results from statistical analyses.

## Data Availability

• Microscopy data reported in this paper will be shared by the lead contact upon request. • This paper does not report novel code. • Any additional information required to reanalyze the data reported in this paper is available from the lead contact upon request.
