# Supplementary material for: Cytoplasmic dynein1 intermediate-chain2 regulates cellular trafficking and physiopathological development in Magnaporthe oryzae
Source: iScience. 2023 Feb 10;26(2):106050. doi: 10.1016/j.isci.2023.106050 (PMC9971887; doi:10.1016/j.isci.2023.106050)
Supplement: Document S1. Figures S1–S3 and Tables S1 and S2 [file mmc1.pdf]

iScience, Volume *26*

Supplemental information

Cytoplasmic dynein1 intermediate-chain2 regulates cellular trafficking and physiopathological development in *Magnaporthe oryzae*

Lily Lin, Ibrahim Tijjani, Hengyuan Guo, Qiuli An, Jiaying Cao, Xiaomin Chen, Wende Liu, Zonghua Wang, and Justice Norvienyeku

Supplemental information

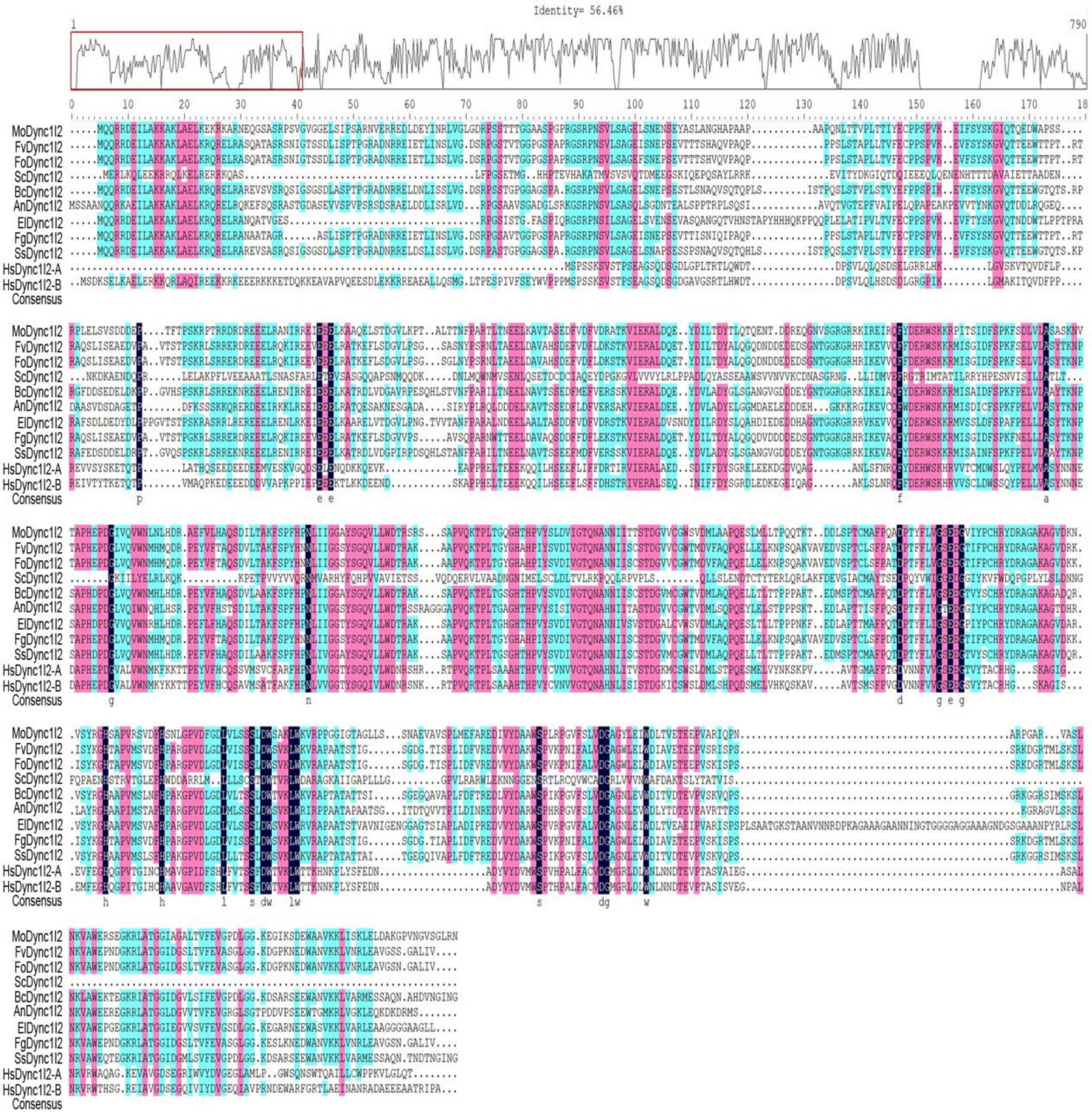

Figure S1. Multiple protein sequences alignments of DYNC1I2 retrieved from *M. oryzae, H. sapiens*, and other selected fungi species. Related to Figure 1. Multiple sequence alignment and homology analyses were performed using DNAMANx software.

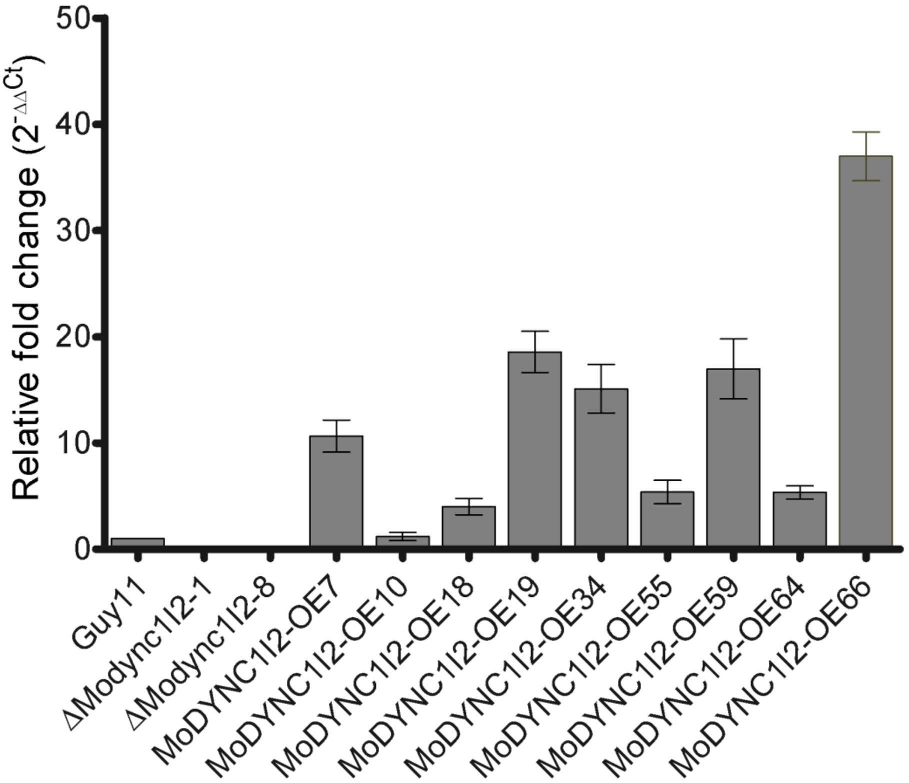

Figure S2. RT-qPCR assisted confirmation of successful deletion of *DYNC1I2* and the expression level of DYNC1I2 in the individual over-expression (OE) strains. Related to Figure 3, Figure 4, and Figure 5.

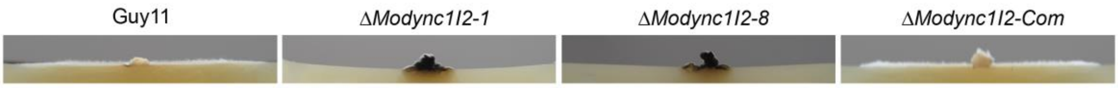

Figure S3. Targeted gene replacement *MoDYNC1I2* attenuate radial development of vegetative hyphae in *M. oryzae.* Related to Figure 3.

Table S1. Annotation for cytoplasmic dynein 1 intermediate chain 2 (Dync1I2) from selected strains used phylogenetic analysis along with their accession numbers. Related to Figure 1 and Figure S1.

Species	Annotation	Gene accession code (ID)
Fusarium verticillioides	FvDync1I2	*FVEG_00889*
Fusarium oxysporum	FoDync1I2	*FOXG_00623*
Fusarium graminearum	FgDync1I2	*FGSG_00428*
Botrytis cinerea	BcDync1I2	*BCIN_02g02260*
Sclerotinia sclerotiorum	SsDync1I2	*SS1G_01856*
Magnaporthe oryzae	MoDync1I2	*MGG_04771*
Eutypa lata	ElDync1I2	*UCREL1_7148*
Aspergillus nidulans	AnDync1I2	*AN1454.2*
Homo sapiens	HsDync1I2-A	*hsa:1780*
Homo sapiens	HsDync1I2-B	*hsa:1781*
Saccharomyces cerevisiae	ScDync1I2	*YDR488C*

Table S2. List of primers and their corresponding nucleotide sequences. Related to STAR Methods.

Name	Primer
Modync1I2-AF	GAACAAAAGCTGGGTGGAACGCAGGTCTGGATGGAT
Modync1I2-AR	CAGCGGCGCGCCGAACGCCGTCCGTACAGTTGAATG
Modync1I2-BF	ACCGGGCCGGCCGGACTTGTCCAGCAACGCAGAGG
Modync1I2-BR	GGTGGCGGCCGCTCTGCACACCAACCGTCCATCAC
Modync1I2-OF	CCGAGTTGAAGGAGAAGAGG
Modync1I2-OR	CAGTGCCGTGGGTTTGA
Modync1I2-UF	TTTCATTACCACGCTACCC
Modync1I2-UR	GCAAAGTGCCGATAAACATA
Modync1I2-COMF	GAACAAAAGCTGGGTCTCTACTCACGGCTGTCGTTC
Modync1I2-COMR	CTGCAGGCATGCAAGGTTCCTCAGGCCAGAGACGCCA
Modync1I2-OEF	AACCCAATCTTCAAACTCGAGATGCAGCAACGCCGAGA
Modync1I2-OER	GCCCTTGCTCACCATAAGCTTTCAGGCCAGAGACGCCA
Modync1I2_pGDG_F	CGGTACCGCGGGCCCG GGATCCATGCAGCAACGCCGAGAT
Modync1I2_pGDG_R	GATTATGATCAGTTA TCTAGATCAGTTCCTCAGGCCAGAGA
Modync1I2-qF	CCAAGTTGTGGAAAGTGCGG
Modync1I2-qR	CGAGAACACACCAGGTCGAA
MoHIS-hypo.-qF	GAAAAAGCCAGTTCCAGGCG
MoHIS-hypo.-qR	GTTCCCCCTTGCCTTCGTTA
MoHis1-like-qF	ACACCTTCGCACCCTACCTA
MoHis1-like-qR	GGGTTGCTCAAACACTCCCT
MohisH3c-like-qF	AAAGTAAAGAAGGCAGGGGC
MohisH3c-like-qR	CGGTGCTCTTTTGGTAACGC
MoHis-H3-qF	GAGGTGTCAAGAAGCCCCAC
MoHis-H3-qR	GAAGACTGGAAGCGGAGGTC
MoKIN1-qF	TTGGTATGAGAGCGAAGTC
MoKIN1-qR	TAATCTGAGTCCTTGCCTTG
MoKIN2-qF	TTCACTCGTCAGCATTCT
MoKIN2-qR	TTGTCTTCTTCGGTATCCTT

Name	Primer
MoKIN-qF	CACATCTCACATCGTCAAG
MoKIN-qR	CCTCCTCTGTCTCATCATC
MoBUF-qF	GCCGACAAGAAGATTACTG
MoBUF-qR	CGCCGTTTGGAATGTATT
MoALB1-qF	TGCCAAGGAGAAGAAGAC
MoALB1-qR	TAAGCGACATCAGCGAAT
MoRSY1-qF	ACTTGCGTCTATGAGTGG
MoRSY1-qR	CATGGTGGTGTCCTTGTA
MoHTF1-qF	AAATGTTATGACGAAAGAGGAT
MoHTF1-qR	TTGTGTTGAAAGTTGAAGTCT
MoHTF4-qF	ATTTCGTGGCTTTCATTAGG
MoHTF4-qR	AGTCTTCGGGTATCATATCC
MoHTF5-qF	TACTGGTGAACGCTATGG
MoHTF5-qR	GTGGTTGGAGTGATCTGAT
MoHTF6-qF	AAGGCAAGGATGTTACGA
MoHTF6-qR	ACCAGGAGCAGTATAAGC
MoTub3-qF	TCTGACTTCAGGAATGGTCGTTAC
MoTub3-qR	AGCGGTCTGGATGTTGTTGG
